# Quantum Limit of Quality Factor in Silicon Micro and Nano Mechanical Resonators

**DOI:** 10.1038/srep03244

**Published:** 2013-11-19

**Authors:** Shirin Ghaffari, Saurabh A. Chandorkar, Shasha Wang, Eldwin J. Ng, Chae H. Ahn, Vu Hong, Yushi Yang, Thomas W. Kenny

**Affiliations:** 1 Stanford University, Mechanical Engineering Department, 440 Escondido Mall, Stanford, CA 94305, USA; 2 Stanford University, Electrical Engineering Department, 350 Serra Mall, Stanford, CA 94305, USA

## Abstract

Micromechanical resonators are promising replacements for quartz crystals for timing and frequency references owing to potential for compactness, integrability with CMOS fabrication processes, low cost, and low power consumption. To be used in high performance reference application, resonators should obtain a high quality factor. The limit of the quality factor achieved by a resonator is set by the material properties, geometry and operating condition. Some recent resonators properly designed for exploiting bulk-acoustic resonance have been demonstrated to operate close to the quantum mechanical limit for the quality factor and frequency product (*Q-f*). Here, we describe the physics that gives rise to the quantum limit to the *Q-f* product, explain design strategies for minimizing other dissipation sources, and present new results from several different resonators that approach the limit.

Micromechanical resonators have become viable timing and frequency references^1^,^2^. Miniaturization and compatibility with electronic fabrication potentially reduce size and cost of attaining high performance on-chip oscillators^3^. Resonators have been realized with ultra-stable frequency^4^ and high quality factor (*Q*) critical for high performance reference oscillators^1^,^3^. High-*Q* performance is limited by the mechanisms that dissipate the mechanical energy of the resonator.

Energy is dissipated in micromechanical resonators through several mechanisms such as air damping, clamping loss and thermoelastic dissipation (TED). These loss mechanisms are essentially classical in nature. Air damping refers to the loss of energy to the air molecules surrounding the resonating structure^5^ and is the dominant energy loss mechanism in low frequency resonators that are not operated in vacuum. Clamping loss is the energy lost to the anchor from a resonator. The energy loss through the anchor depends on the design of the stem connecting the resonator to the anchor^6^,^7^,^8^,^9^,^10^, and is usually mitigated by symmetric operation of multiple elements such that the forces and moments at the anchor sum to zero. TED^11^,^12^ is a coupled thermo-mechanical phenomenon, wherein strain-induced temperature gradients induce thermal transport and energy loss. Though the origin of TED can be traced back to phonon interactions, it is possible to model this effect purely based on classical heat transfer and the resulting entropy generation^12^. For this set of mechanisms (air damping, anchor loss and TED), the total energy dissipation can be significantly reduced by appropriate design of the resonator and operation in vacuum. Another energy loss mechanism – described as the Akhiezer effect (AKE) – arises from quantum mechanical phonon processes and presents a fundamental upper limit to the *Q-f* product for resonators^13^, depending only on the properties of the resonator material.

“Quantum energy dissipation” in vibrating solids arises due to scattering (AKE) and transport (TED) of phonons. To understand the fundamentals of these processes, we begin by considering a crystalline solid at room temperature. The solid may be represented by an array of atoms held near their equilibrium positions by interatomic forces; this array of atoms has quantized vibrational modes referred to as phonons^14^. Phonons have wavelength (*λ*), energy (ω), and momentum (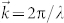); the relationship between the frequency of the mode and the wavelength of the mode is referred to as the dispersion relation 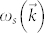, and is approximately linear for small values of ω and 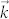.

At equilibrium, phonons modes are populated according to the Planck distribution. If the entire solid is subjected to a longitudinal elastic vibration, such as occurs during operation of a micromechanical oscillator, the periodic distortions of the solid are represented by a local variation in the dispersion relation; in effect, extension of the crystal reduces the slope of the dispersion relation, while compression of the crystal increases the slope of the dispersion relation. The fractional change in the slope of the dispersion relation per unit change in volume is referred to as the Grüneisen parameter^15^,^16^, 
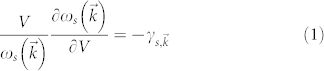
where the indices for the Grüneisen parameter take into account the details associated with the mode shape and orientation with respect to the crystal^16^. During such a distortion of the crystal, the population of the phonon modes no longer matches the Planck distribution function, and inelastic phonon scattering processes act to redistribute the population, thereby approaching a new thermal equilibrium with the Bose-Einstein distribution.

There are 3 timescales that determine the outcome of the scattering processes – the vibrational period of the lattice distortion (*τ_v_*), the mean scattering time (*τ_s_*), and the relaxation time for thermal transport between regions of different lattice distortion (*τ_th_*). For silicon resonators, the mean scattering time (*τ_s_*) is a few picoseconds^17^.

In cases where *τ_v_* > *τ_th_* > *τ_s_*, (true for most bending-mode MEMS resonators) the scattering process leads to establishment of a new thermal equilibrium at a different temperature (cooler for extension, warmer for compression), and thermal transport can take place between regions with different strain. Because the transport is irreversible, entropy is generated and energy is dissipated – this phenomenon is described as Thermoelastic Dissipation (TED), and can dominate for resonators that have significant strain gradients, such as for bending modes of a beam^18^. To avoid TED one can select resonator designs that will not exhibit significant strain gradients, such as extensional modes of rings, disks and bars^13^,^19^.

For resonators that vibrate without producing strain gradients, and when *τ_v_* > *τ_s_*, the phonon scattering process leads to establishment of a new population distribution representing a new temperature. During this process, entropy is increasing as always happens during evolution towards thermodynamic equilibrium^14^, and energy is being dissipated; this phenomenon is described as the Akhiezer Effect (AKE)^20^,^21^,^22^,^23^,^24^. For very high-frequency resonators when *τ_s_* > *τ_v_*, there is insufficient time during a period of vibration for scattering to alter the distribution of phonons in the crystal. In this very high-frequency case, AKE should be suppressed, and the *Q-f* product may exceed the normal Akhiezer limit^24^,^25^,^26^,^27^.

## Results

The dynamics of the phonon-phonon interactions are captured by the Boltzmann transport equation (BTE)^15^. 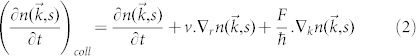
where 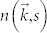 is the distribution function of phonons with wavevector 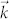 and polarization *s*, *v* = *∂ω*/*∂k* is phonon group velocity and *F* is an external force field. 
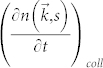
 is the rate of change in phonon population due to phonon-phonon collisions.

The spatial terms in the BTE become negligible for cases where: (i) the spatial change in the phonon distribution is insignificant due to small strain gradients in the resonating solid, and (ii) *F* is zero for a simple elastic crystal with periodic boundary conditions. (i) is valid for many micro/nano mechanical resonators operated without strain gradients, such as in the Lamé mode resonator and contour mode of a ring resonator, and when these resonators are operated in ordinary environments. The resonators are operated in the natural vibration mode of the structure. If surface effects can be neglected (as for high quality crystalline silicon which is employed in our resonators), the solid boundaries are strain free and vibration corresponds to a standing wave, which defines the mode shape. Periodic boundary conditions are appropriate for standing wave solution of the BTE. This assumption is valid when the anchors are carefully designed so not to interfere with the mode symmetry (placed at node points and displacement gradient in the anchor is negligible to first order). In this case, the pertinent BTE reduces to^21^: 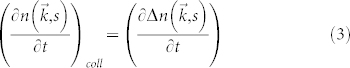
where 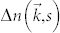 is the perturbation from thermal equilibrium in phonon population distribution at the thermal reservoir temperature.

We apply a relaxation time approximation to the collision term and assume phonon population decays toward a Bose-Einstein distribution 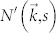
21. The relaxation time approximation signifies phonon scattering in a vibrating solid in the limit of absent spatial dispersion. This assumption is necessary to derive the attenuation of a standing vibration wave in a solid. 
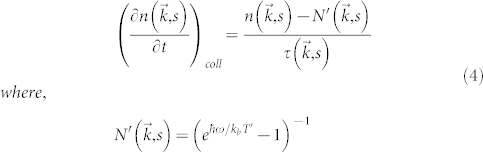
Here, 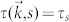 is the relaxation time of the phonon scattering, i.e. mean time between collisions, which is often condensed into a single time constant *τ* independent of wavevector 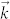 and polarization *s*, and 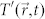 is modulated local temperature.

Assuming a harmonic solution to equation (3) (Δ*T*, Δ*n*, Δ*ω* ~ *e^j^*^2*πft*^) with the mechanical resonance frequency *f*, equation (4) can be solved for *n*^22^. 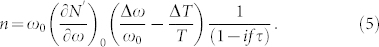
where a Taylor expansion of the term 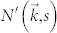 was used and the subscript 0 denotes equilibrium. The average rate of energy dissipated by phonon collisions is proportional to second order of strain; therefore, to first order, energy must be conserved. 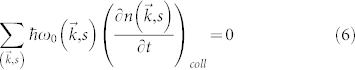
The local temperature change may be derived to satisfy the energy conservation as Δ*T*/*T* = (Δ*ω*/*ω*_0_)*I*_00_/*I*_01_ and substituted in equation (5) for *n*. *I*_00_(*fτ*) and *I*_01_(*fτ*) are angular integrals^21^. Here, it is assumed that the frequency shift is the same for all phonon modes, which is supported by neglecting the spatial terms in BTE. This assumption is specific to the AKE limit.

*n* is complex in general, and its imaginary part corresponds to the attenuation (Γ) of the harmonic vibration of the solid^20^,^23^ as given by^21^, 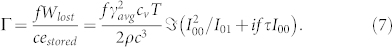
where *W_lost_* is the local energy lost per unit volume, *e_stored_* is energy stored per unit volume, *γ_avg_* is average Grüneisen's parameter, *ρ* is the density of the solid and *c_v_* and *c* are the heat capacity per unit volume and average acoustic velocity respectively.

In the AKE limit (*fτ* ≪ 1), the imaginary part may be simplified to give, 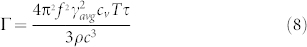
Using the attenuation relation above, the total energy lost in the resonator is estimated as, 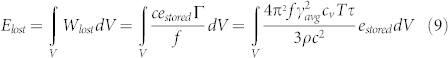
Thus, the quality factor of the micro- (nano-)resonator can be estimated as 
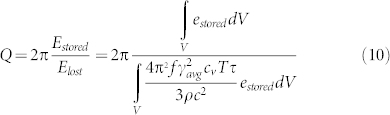
We see that all the details related to the vibration mode shape and volumetric change are represented in the integrals in the expression above, and that those integrals perfectly cancel out, leaving only constants. Consequently, in homogeneous micromechanical resonators, the AKE limit of quality factor is simply given by 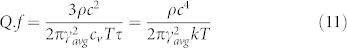
where *k* = 1/3*c_v_c*^2^*τ* is the thermal conductivity of the solid.

Table 1 lists some of the commonly used resonator materials and their *Q-f* product limits together with the Grüneisen parameter used to determine these limits. Amongst all, SiC has exceptionally high *Q-f* product.

To obtain the *Q-f* products of anisotropic silicon we use the first expression in equation (11) and the expression for *c_v_*^15^ that uses the transverse wave velocity in silicon for the correct evaluation of the Debye temperature. This more accurate accounting for *c_v_* yields a somewhat smaller *Q-f* value than some previous results^13^ and was employed in^24^.

Maris^23^ provides a more complete analysis, including the elastic and inelastic collision dynamics, and finds an expression for *Q* in this limit with an additional term that relates the period of the oscillation to the timescale for phonon scattering, 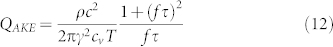
Equation (12) reduces to equation (11) with the assumption invoked throughout this paper that the frequency of vibration of the solid is low enough to allow the phonons to interact and reach a new equilibrium, i.e. *fτ* ≪ 1.

## Discussion

This upper limit set by Akhieser effect on quality factor in dielectric micro/nano mechanical resonators may be represented by a boundary in the *Q-f* plane. The region below the AKE limit line is accessible for resonators of the same material.

**Figure 1:**
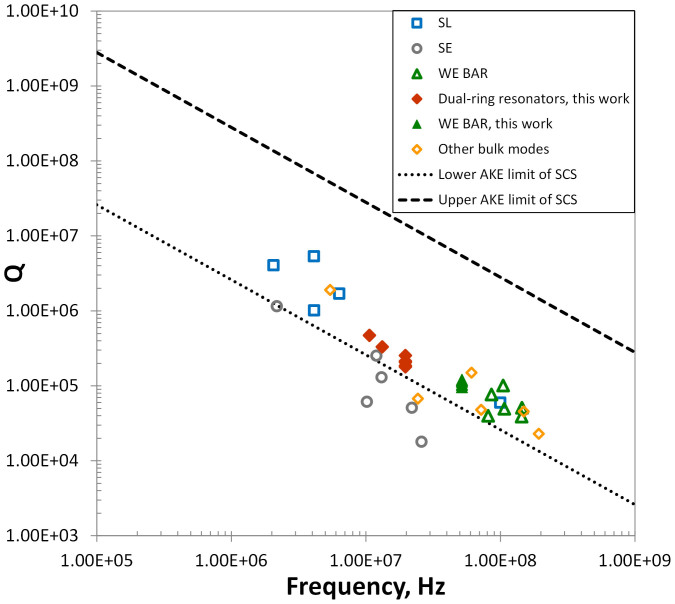
Quality factor and frequency of recently reported high frequency silicon bulk resonant mode devices. Plot shows our fabricated dual ring resonators (filled diamonds) resonating in contour bulk (breathe) mode and width extensional bulk acoustic resonators, WE BARs, (filled triangles) in the acceptable AKE region. SL refers to square Lamé mode resonators of Refs. 8,28,29,30,31. SE refers to square extensional mode resonators of Refs. 32,33,34,35. Open triangle WE BARs refer to WE resonators of Refs. 19,36,37,38. The other bulk mode resonators represent disk resonators of Refs. 9,39,40,41,42,43. The resonance mode shapes for the relevant resonators are shown in Figure 2. Upper and lower limit of AKE are derived from equation (11) for corresponding value of the Grüneisen's parameter for Si: 0.17 and 1.5.

Figure 1 compares some of the state of the art devices and recent devices from our group in this AKE limit for silicon resonators. To our knowledge, there are no examples of silicon resonators with *Q-f* products that exceed this limit.

To approach the AKE limit in resonators, other damping mechanisms must be minimized or eliminated altogether. Vacuum packaging or operation in vacuum is necessary for elimination of energy loss to surrounding air molecules. Design of symmetric resonators that greatly reduce the strains present at the anchors is necessary to reduce energy loss due to anchor damping. Selection of resonator modes that do not exhibit strain gradients (such as extensional modes) are important for suppression of TED. The Lamé modes of squares and contour modes of rings are representative examples of low-TED modes because the total strain distribution is uniform.

Strain gradient pertains to nonzero spatial gradients in the Boltzmann transport equation that cause thermal transport. Heat transfer drives the irreversible energy loss. While under the assumption of periodic boundary condition the AKE limit of energy dissipation is the same for all Si resonator modes, it is expected that for devices where *Q*(TED) prediction is comparable to or lower than *Q*(AKE), significant spatial gradients introduce the thermal transport relaxation time *τ_th_* into the model that may further reduce the quality factor by the mechanism of TED.

**Figure 2:**
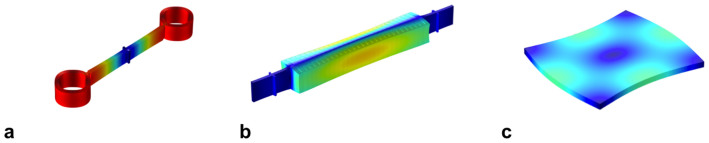
(a) Finite-element method (FEM) simulation of bulk mechanical mode shapes for (a) dual ring resonator, (b) Width extensional (WE) BAR, and (c) Lamé mode square colored with total displacement relative to the maximum displacement of the dual ring resonator. Color intensity corresponds to maximum (compressive) displacement where red and minimum displacement where deep blue.

**Figure 3:**
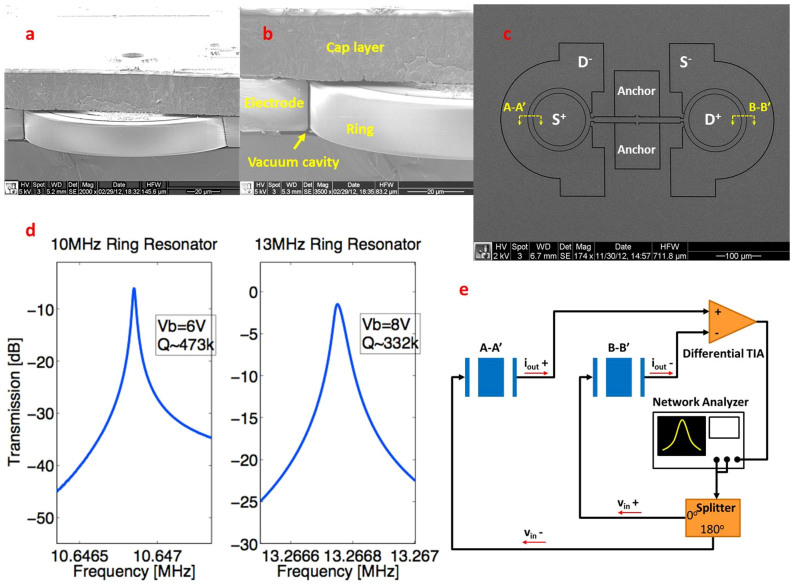
Frequency response measurements of dual ring resonators. (a) SEM image of one ring inside the encapsulation package. (b) A zoomed in view of the ring, outer drive/sense electrode and cap layer, capacitive transduction is established in the vacuum cavity. (c) A top view SEM image of the dual ring resonator geometry showing the ring pair connected by the coupling bar and anchored off the center of the bar. The Drive/Sense (D/S) electrodes are labeled as in a typical measurement setup shown in (e). (d) Transmission response of the resonators near 10 MHz and 13 MHz, measured with the setup shown in (e). (e) Schematic of the experimental setup. Each ring pair is depicted in the cross sectional view as marked in (c). A differential drive voltage is applied to each drive electrode generates an out of phase sense current with respect to the input voltage. The two opposite sense currents are added by the differentiated in the TIA, while their unsigned noise is subtracted out.

Our group has been focused on characterization and optimization of dissipation in MEMS resonators. To this end, we have developed a wafer-scale vacuum encapsulation process to eliminate damping from air molecules^5^, and we have focused on device designs with TED optimization^18^. More recently, we have developed a series of ring-extensional mode resonators in this process that suppress TED and allow these devices to approach the AKE limit. With comparable frequencies, the *Q*'s of these recent resonators exceed those of most previously published bulk mode resonators. These resonators consist of a pair of symmetric rings driven into an in-plane “breathing” mode, i.e. extension of the ring contour, which is free of strain gradients. These rings are coupled to a nodal anchor point by a bar whose in-plane fundamental extensional mode is matched to the modes of the rings, as shown in Figure 2. Within the tolerances of our fabrication process, the ring and bar extensional modes become coupled into a single high-*Q* resonator mode. The mechanical resonance is driven and sensed by capacitive transduction by applying an AC polarization voltage to the drive electrodes. The particular electrode configuration shown in Figure 3 enables efficient transduction of the contour (extensional) mode of the rings as they expand and contract simultaneously. The resonant frequency of this mode is a sole function of the average ring radius, *R*, 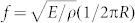 for homogenous material properties where *E* is the Young modulus. The asymmetric drive/sense layout of the rings is characteristic of differential transduction, which allows application of symmetric forces and subtraction of parasitic capacitive signals for improved accuracy in the measurement. Representative frequency response of these resonators near the resonance is shown in Figure 3d.

As shown in Figure 1, these resonators achieve quality factors higher than previously published resonators in 10–20 MHz range.

**Figure 4:**
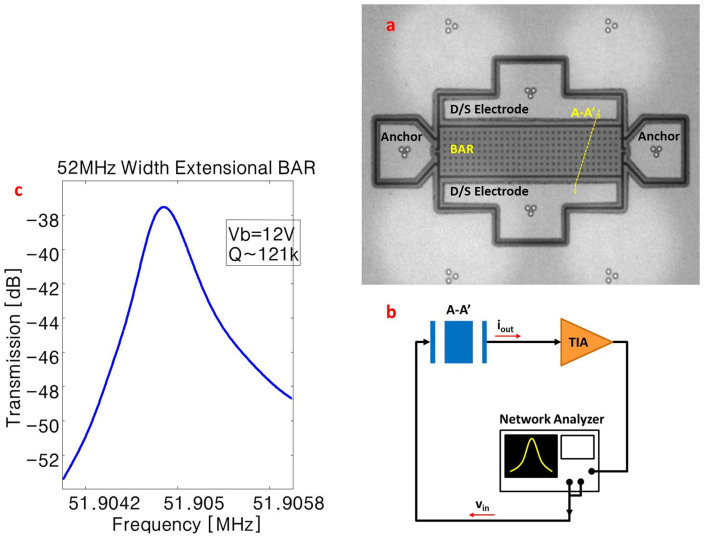
Frequency response measurement of width extensional (WE) BARs. (a) Top view SEM image of WE BAR and actuation electrodes. The (D/S) electrodes are labeled as in a typical measurement setup either one can be used as the drive (D) electrode and the other as sense (S) electrode. (b) Schematic of the experimental setup. (c) Transmission response of the resonator near 52 MHz.

In addition, we have designed and fabricated a second set of high frequency resonators in Figure 1 and refer to these as Width Extensional Bulk Acoustic Resonators (WE BAR). As shown in Figure 4, WE BAR consists of a rectangular bar that resonates by expanding and contracting its width. Similar to the dual ring resonator, the WE BAR is transduced by the parallel electrodes along the length of the bar. *Q-f* products close to the AKE limit are observed for these devices.

Figure 2 compares the simulated mode shapes of these resonators where the deformations are scaled to the maximum deformation of the dual ring resonator. The deformation is nearly uniform in the rings and the deformation gradient in the WE BAR and the Lamé mode square is significantly lower than the dual ring resonator. This indicates TED is less significant for the latter two. Quantitative results from fully coupled TED simulations of an example dual-ring resonator and a WE BAR are compared in Table 2 and show *Q*(TED) of the WE BAR is higher by a factor of 7. Because TED is suppressed, the WE BAR operates closer to the AKE limit.

We mentioned that because of the absence of large strain gradients associated with the vibrational mode, the contour mode of dual ring resonators and width extension of the bar should be free of TED for homogeneous material properties. However the intrinsic anisotropy of the materials properties of SCS causes very slight nonuniformities in the volumetric strain around the ring, which is due to anisotropic stiffness. By examination of Figure 2, largest gradients are found in the bar that connects the rings to the anchor at the node point of the bar extension. Because of this strain gradient, there is some energy loss from TED, contributing to reductions in the total *Q-f* product for these resonators to around 20% of the AKE limit. For an example 20 MHz resonator, as reported in Table 2, the measured *Q* was 255 × 10^3^, whereas *Q*(TED) from simulation was 337 × 10^3^ and calculated *Q*(AKE) was 1.17 × 10^6^. The reduction of the measured *Q* by a factor of 4.5 relative to the AKE limit arises due to residual TED in these resonators. The total *Q* from independent contributions of AKE and TED is 1/(Q(TED)^−1^ + Q(AKE)^−1^) = 261 × 10^3^ which indicates that Q is almost fully contained by the contributions of AKE and TED.

It is important to note that the AKE limit line was derived for an average Grüneisen's parameter. For a more accurate prediction, we would need to take into account the material anisotropy and its effect on phonon scattering time; the same consideration applies to the thermal phonon time constant, *τ*. We notice that the variations in the reported values for the Si Grüneisen's parameter may arise from different accounting for the anisotropic material properties; the distribution in estimates for the Si Grüneisen's parameter lead to published estimates for the AKE limit that range over three times the average^24^. We represent this variation as a set of upper and lower AKE lines in Figure 1. For different resonators, such as rings and bars, which can exhibit strains along different directions in the SCS crystal, the correct choice of parameters for calculating the AKE limit can be different within this range. Aside from this modest interaction between the mode shape and the crystal geometry, we find that general “AKE limits” can be considered as accurate, and that designers working to optimize *Q* for resonators should be aware of the implications of this limit.

## Author Contributions

S.A.C., S.G. and T.W.K. conceived the study. S.W., E.J.N., C.H.A., V.H., and Y.Y. fabricated the devices. S.W., S.G. and E.J.N. performed experiments. S.G. interpreted the model, analyzed the data and carried out FEM simulations. S.G., S.A.C. and T.W.K. wrote the manuscript.

## Figures and Tables

**Table 1:** Akhiezer limit of *Q-f* product for common resonator materials and the corresponding average Grüneisen parameter

Material	*Q-f* (× 10^−13^)	*γ_avg_*
Si	2.3	0.51
Quartz	3.2	0.87
AIN	2.5	0.91
Diamond	3.7	0.94
Sapphire	11.3	1.1
SiC	64	0.3

**Table 2:** Comparison of experimentally measured quality factor (*Q_meas_*) for a dual ring resonator and a WE BAR with *Q* calculated from Eq. 11 in the Akhiezer limit (*Q_AKE_*) and *Q* from fully coupled finite-element method (FEM) thermoelastic simulation (*Q_TED_*). Note that *Q_AKE_* signifies an average upper bound to attainable *Q* by phonon scattering and is derived independent of the resonant mode shape. The average Grüneisen parameter for Si is used

Device	*f* (MHz)	*Q_AKE_* (×10^−4^)	*Q_TED_* (×10^−4^)	*Q_meas_* (×10^−4^)
Dual Ring Resonator	19.7	117	33.7	25.5
WE BAR	50.2	117	247	10.5
